# Improvement of plantar hyperhidrosis following the treatment of primary palmar hyperhidrosis with botulinum toxin injection

**DOI:** 10.1111/jocd.16418

**Published:** 2024-05-31

**Authors:** Fatemeh Moieneddin, Saba Hasanzadeh, Martin Kassir, Reza M. Robati

**Affiliations:** ^1^ Skin Research Center Shahid Beheshti University of Medical Sciences Tehran Iran; ^2^ Worldwide Laser Institute Dallas Texas USA; ^3^ Department of Dermatology Loghman Hakim Hospital, Shahid Beheshti University of Medical Sciences Tehran Iran

## ETHICS STATEMENT

The ethical issues were completely considered to prepare this case report according to our institution’s ethical board guidelines. Moreover, this article was prepared regarding the declaration of Helsinki.

Dear Editor,

Primary hyperhidrosis is characterized by an abnormal increase in sweating of the entire body or a localized region. Patients with this condition experience emotional and psychological distress, leading to a reduced quality of life when compared to healthy individuals.^1^ The etiology of this condition is not well understood; however, it is believed that dysfunction in the autonomic nervous system may play a role. Another possible mechanism is dysfunction in the central control of emotions which involves the control of the sweat center of the hypothalamus by the cortex instead of the normal thermoregulatory inputs.^2^ Subcutaneous botulinum toxin (BT) injection is a noteworthy therapeutic option as it blocks the release of acetylcholine, a neurotransmitter that triggers the eccrine glands to discharge sweat. This treatment method can safely result in a long‐lasting reduction in sweat secretion in the axillary region. BT is also used offlabel for hyperhidrosis of the craniofacial and inguinal regions, as well as the palms and soles.^3^

Four patients, aged 18–26, visited our clinic with primary palmar and plantar hyperhidrosis. Among the patients, three were female and one was male. The iodine‐starch test was performed in both hands and feet before the injection for each patient which revealed severe hyperhidrosis in both areas. The injection of 250–300 units of abobotulinum toxin A (Dysport®; Ipsen Biopharmaceuticals, Wrexham, UK) into both palms (no injections into the soles) was performed with the standard protocol. The injection sites were 1 cm apart from each other and 3 units of BT were injected into each site. We observed a significant improvement in both palmar and plantar hyperhidrosis in a week following the BT injection. The iodine‐starch test was repeated, showing negative results for the hands and nearly completely negative results for the feet in each patient, with similar effects observed on both sides (Figure 1). In addition, the two patients suffering from excessive axillary sweating reported a reduction in sweat secretion following palmar injections. There were not any significant side effects except for mild and temporary weakness of the flexion and rotation of the hand fingers in two patients. The improvement of hyperhidrosis persisted during the 3‐month follow‐up period.

**FIGURE 1:**
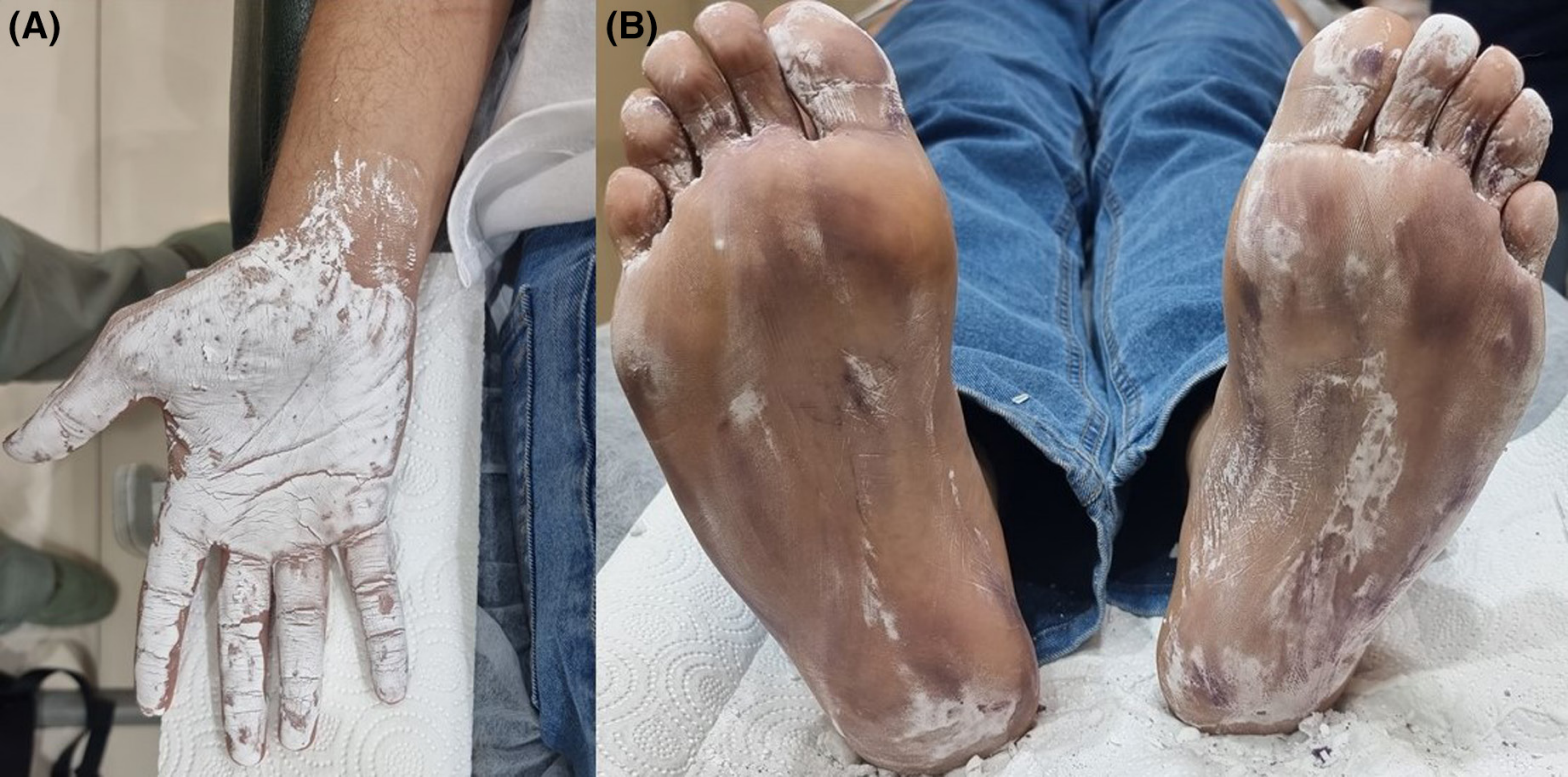
The patient's right hand with negative iodine test a week after BT injection in palms (A), the patient's plantar negative iodine test a week after BT injection in palms (B).

To explain this outcome, we propose that changes in emotional status may play a role. The hypothalamus and limbic system, which are components of the central nervous system, regulate sweat secretion. Efferent nerves originating from the hypothalamus thermoregulatory center pass through the spinal cord and synapse in the sympathetic ganglia. From there, postganglionic sympathetic nerves innervate the eccrine glands and stimulate M3‐muscarinic receptors by releasing acetylcholine. During heightened emotional states, the limbic system descends efferent nerves through the ventral horn of the spinal cord. These nerves then synapse with sympathetic neurons located in the nucleus intermediolateralis. The sympathetic neurons, in turn, innervate the adrenal gland, triggering the release of catecholamines into the bloodstream which leads to the secretion of sweat from both eccrine and apocrine glands.^4^

Injecting BT into the palms of the hands effectively inhibits the activation of the eccrine glands by acetylcholine, leading to a notable reduction in sweat secretion from the palms.^3^ Consequently, this intervention reduces the anxiety, stress, and emotional burden associated with hyperhidrosis in affected individuals. The administration of BT also results in a decrease in the release of catecholamine, which can be attributed to the reduction in efferent pulses from the limbic system.^4^ This decline in catecholamine release subsequently diminishes the stimulation of adrenergic receptors in both the eccrine and apocrine glands.

Our observations indicate that the local injection of BT might indirectly influence the amount of sweat secreted by other areas of the body. According to the limitation of this study, additional research with a large sample size and a long follow‐up period is required to elucidate this possible association more definitely. However, it could be recommended to avoid BT injections in both the palmar and plantar regions in the same session. This is due to the potential indirect effects of the BT injection, which may sufficiently improve symptoms and potentially require lower doses of BT for subsequent treatment of the other areas. Additionally, this approach has the potential to avoid the extreme pain of BT injection in the sole and minimize the occurrence of adverse reactions.

## FUNDING INFORMATION

This article received no funding source.

## CONFLICT OF INTEREST STATEMENT

None.

## Data Availability

The data that support the findings of this study are available on request from the corresponding author. The data are not publicly available due to privacy or ethical restrictions.
